# Parvovirus B19 in Rheumatic Diseases

**DOI:** 10.3390/microorganisms12081708

**Published:** 2024-08-19

**Authors:** Rosaria Arvia, Maria A. Stincarelli, Elisabetta Manaresi, Giorgio Gallinella, Krystyna Zakrzewska

**Affiliations:** 1 Department of Experimental and Clinical Medicine, University of Florence, 50134 Florence, Italy; mariaalfreda.stincarelli@unifi.it (M.A.S.); krystyna.zakrzewska@unifi.it (K.Z.); 2 Department of Pharmacy and Biotechnology, University of Bologna, 40126 Bologna, Italy; elisabetta.manaresi@unibo.it (E.M.); giorgio.gallinella@unibo.it (G.G.); 3 S. Orsola-Malpighi Hospital—Microbiology, 40138 Bologna, Italy

**Keywords:** human parvovirus B19, B19V, rheumatologic diseases, arthritis, systemic sclerosis, systemic lupus erythematosus

## Abstract

Parvovirus B19 (B19V) is a human pathogen belonging to the *Parvoviridae* family. It is widely diffused in the population and responsible for a wide range of diseases, diverse in pathogenetic mechanisms, clinical course, and severity. B19V infects and replicates in erythroid progenitor cells (EPCs) in the bone marrow leading to their apoptosis. Moreover, it can also infect, in an abortive manner, a wide set of different cell types, normally non-permissive, and modify their normal physiology. Differences in the characteristics of virus–cell interaction may translate into different pathogenetic mechanisms and clinical outcomes. Joint involvement is a typical manifestation of B19V infection in adults. Moreover, several reports suggest, that B19V could be involved in the pathogenesis of some autoimmune rheumatologic diseases such as rheumatoid arthritis (RA), juvenile idiopathic arthritis (JIA), systemic sclerosis (SSc), systemic lupus erythematosus (SLE), or vasculitis. This review provides basic information on the B19 virus, highlights characteristics of viral infection in permissive and non-permissive systems, and focuses on recent findings concerning the pathogenic role of B19V in rheumatologic diseases.

## 1. Introduction

Parvovirus B19 (B19V) is a human pathogenic virus in the *Parvoviridae* family [1], widely diffused in the population and responsible for an ample range of diseases, diverse in pathogenetic mechanisms, clinical course, and severity [2,3]. Normally transmitted via the respiratory route, infection is typical for the specific tropism of B19V towards erythroid progenitor cells in the bone marrow, leading to cell apoptosis, and for its intense viremia that allows for systemic diffusion to disparate tissues. In the initial phase, the effect of the virus is a temporary block of erythropoiesis, that can be clinically evident as a severe and refractory aplastic crisis in case of underlying hematological disorders. In a second phase, the virus is responsible for the characteristic, mild erythematous rash of the fifth disease more frequently observed in children, and of more aggressive arthralgias typical in elder subjects. The immune response is normally capable of neutralizing viral infectivity and relieving the block in erythropoiesis, while it can contribute to the second phase of the disease, which occurs in the presence of an antibody response, supposedly by deposition of immune complexes. On the other hand, an impaired immune response unable to effectively neutralize the virus may lead to chronic infections, possibly manifesting as chronic anemia, a condition observed in cases of congenital or acquired immunodeficiencies, in case of concurrent onco-hematological disorders, or linked to immunosuppression therapeutic regimens [4]. Finally, B19V can cross the placental barrier, thus posing a risk of intrauterine infections with possible effects on the fetuses. Fetal complications range from transitory fetal anemia and nonimmune fetal hydrops to miscarriage and intrauterine fetal death, with an overall risk of adverse fetal outcome that has been reported from 3% to 12%, depending on the gestational age [5]. Understanding the viral lifecycle and impact on host cells, the pathogenetic mechanisms, and the interaction with the immune system, coupled with increased diagnostic awareness, can lead to better management of infections, not least the development of more specific and effective antiviral strategies. The aim of this review is to provide basic information on the virus, highlighting characteristics of viral infection in permissive and non-permissive systems, and to focus on recent findings concerning the pathogenic role of B19V in rheumatologic diseases.

## 2. Biological Characteristics and Epidemiological Features of Parvovirus B19

**Figure 1:**
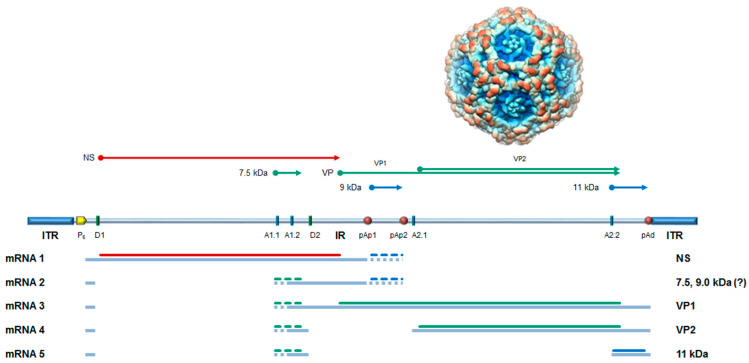
B19V capsid and genome organization. Top coding sequences for the viral proteins. NS, non-structural protein; VP, structural proteins, colinear VP1 and VP2, assembled in a T = 1 icosahedral capsid (above); 7.5 kDa, 9.0 kDa, 11 kDa: minor non-structural proteins. Center: Schematic diagram of B19V genome. ITR, inverted terminal regions; IR, internal region; cis-acting functional sites: P6, promoter; pAp1, pAp2, proximal cleavage–polyadenylation sites; pAd, distal cleavage–polyadenylation site; D1, D2, splice donor sites; A1.1, A1.2, A2.1, A2.2, splice acceptor sites. Bottom: Simplified transcription map of B19V genome, indicating the different mRNAs groups (1–5) with exons and alternative splicing/cleavage forms (dashed), and their coding potential. Modified from [3]. Capsid shell image from PDBJ (www.pdbj.org, accessed on 20 March 2009; EMD-1467) © Protein Data Bank Japan (PDBj) licensed under CC-BY-4.0 International.

B19V shares structural features of the family *Parvoviridae* [2]. A single-stranded DNA (ssDNA) molecule of either polarity is encapsidated in an icosahedral capsid shell, T = 1 arrangement, about 25 nm in diameter (Figure 1). The genome is composed of an internal unique region, coding for all viral proteins, flanked by two inverted, repeated terminal regions. These present palindromic sequences that can fold back into hairpin structures, serving as primers for second-strand synthesis in the replication of the viral genome, via double-stranded DNA (dsDNA) replicative intermediates and a rolling hairpin mechanism. Viral proteins include the non-structural NS1 protein, essential for viral replication and responsible for modulation of the host–cell environment, and other less characterized non-structural proteins (7.5, 9.0, 11.0 kDa) [6]. The viral capsid proteins VP1 and VP2 are translated on the same frame, constituting about 5% and 95% of the capsid subunits, respectively. The VP1/2 common region constitutes the core capsid shell [7]; the unique N-terminus of VP1, VP1u region, is located close to the penton vertex in submolar amounts [8], is responsible for binding to a cognate cell receptor and presents a constituent phospholipase activity [9], both required for viral infectivity.

Circulation of the virus in the human population is global, although with different reported rates in different geographical areas. In temperate areas, epidemic cycles occurring every 3–4 years are typical, and transmission is common in spring-early summer. Infection, as determined by age-specific force-of-infection estimates, is more frequently acquired in childhood and early adulthood, but can occur until elder ages, reaching final seroprevalences that may be higher than 80% [10]. While the infection is normally perceived as mild and clinically uneventful, B19V constitutes a relevant cause of disease that should not be overlooked or underestimated, given the wide circulation of the virus and its intrinsic pathogenic potential. The outcome of infection will be the result of the interplay of viral properties, the underlying genetic and physiological background of the host, and the efficacy of a specific immune response. As a consequence, B19V is implicated in a wide range of diseases affecting different systems, sometimes in very atypical presentations [Table 1].

A compilation of diseases and clinical manifestations associated with parvovirus infection. Frequent clinical manifestations are typical of B19 infection, although the presentation and the course of the disease may vary depending on the physiological and immune status of the individual. Sporadic clinical manifestations include diseases that have been more rarely associated with B19 infection, occurring at a low frequency and without any predictable concurrent factor. The list of sporadic clinical associations is not exhaustive. From [11], modified.

## 3. Infection in Erythroid or Non-Erythroid Cells

B19V shows a striking, selective adaptation to erythroid cells, the only population that can actively sustain a productive viral replication [12]. On the other hand, B19V can become internalized in a wide set of different cell types, normally non-permissive but possibly susceptible to a viral-induced dysregulation of the normal physiology [13]. A difference in the characteristics of virus–cell interaction may translate into different pathogenetic mechanisms and clinical outcomes. Understanding the regulation of viral replication and expression in different cell types is thus a basis for any pathogenetic and clinical assessment and for the individuation of effective therapies and antiviral strategies, still an unmet need.

A productive replicative cycle occurs in erythroid progenitor cells, distributed in the bone marrow or, during fetal life, in the liver or bone marrow depending on gestational age. On histologic examination, B19V-infected bone marrow can show erythroid hypoplasia, and the presence of characteristic enlarged cells, so-called giant pronormoblasts, considered pathognomonic for B19V [14]. Studies on B19V virus–cell interaction are now mostly conducted in in vitro differentiated erythroid progenitor cells (EPCs), easily obtained from peripheral blood [15,16]. Circulating progenitor cells can be expanded and differentiated by stimulation with growth factors, including Erythropoietin (Epo) which is necessary for productive replication [17]. EPCs show different levels of susceptibility and permissiveness to viral replication, depending on the differentiation stage, replicative rate, and physiological conditions [18]. Susceptibility to infection increases with the differentiation state starting from early days, while cellular permissiveness reaches a maximal degree at 6–9 days of differentiation, corresponding to the normoblast stage, after which cells stop supporting effective viral replication; in addition to Epo, hypoxia (1% O_2_), a physiological condition in the bone marrow, increases viral replication [19].

Such a selective tropism towards erythroid progenitor cells and the fine regulation of viral replication are still a matter of investigation, although several key steps have been identified. Firstly, the interaction of the viral particles to the neutral glycolipid globoside, present in abundance on erythroid progenitor cells as well as on the erythrocyte membrane, where they constitute the P antigen [20]. Globoside has been for a long time considered the primary attachment receptor for virions, whose presence is an absolute requirement for a productive replicative cycle [21]. However, experimental data on the physical interaction of B19V capsid and globoside are controversial, and more recent data indicate that a functional interaction can occur within the acidified environment of endosomes, necessary for the subsequent steps of nuclear translocation and the onset of macromolecular syntheses [22,23].

In addition to interaction with globoside, it has been known for a long time that further interaction with a cellular co-receptor(s) is necessary for virus internalization and the onset of a productive replicative cycle. Initial identification of α5β1 integrin as the coreceptor [24] has been questioned. In fact, the N-terminus of the VP1u region has been shown to interact with a specific partner present on the cell membrane of EPCs, and also of a few cell lines able to support viral replication, different from integrin α5β1 [25,26,27]. The viral receptor-binding domain (RBD) has been mapped to aa. 29–130 of the VP1 protein; this region has not been solved in the crystallographic structure of the capsid because of its submolar abundance and possible intrinsic disordered state, although it is predicted to have three α-helix domains. In the native virions, it may be partially buried close to the penton vertex, while its accessibility may be increased by conformational modifications, possibly induced by the interaction of virions with globoside present in the cell membrane [28]. The interaction is specific, as shown by competition experiments, and subject to neutralization by antibodies. The elusive identity of the receptor is still a matter of research; the cellular transmembrane protein AXL has been indicated as a possible candidate [29], necessary not only for the internalization of the virus but also for the onset of a productive replicative cycle, although its distribution within tissues is not as restricted as the specific RBD interaction moiety appears to be.

These initial steps are all necessary for internalization in a very specific cellular environment, to which B19V becomes adapted in a very efficient and restricted pattern. Several levels of restriction to a productive replicative cycle have been identified, starting from the conversion of the incoming ssDNA into a dsDNA replicative intermediate [30], to the close coordination of replication of the viral genome and regulation of transcription and mRNA processing [31]. In the first instance, nuclear translocation of the incoming virions and progressive uncoating of the ssDNA genome, which is completed in the nucleus, create the condition for a second strand synthesis, likely operated by the cellular machinery as a DNA damage response (DDR). Conversion of the ssDNA into a dsDNA is a requirement for the onset of viral macromolecular synthesis and constitutes a relevant restriction step in non-permissive cellular environments. Concerning replication of the viral genome, it occurs by a rolling hairpin mechanism [12], requiring a basic cellular DNA polymerase complex [32], in cooperation with the viral NS1 protein [33] and the cellular pSTAT5, itself a product of Epo signaling [34]. NS1 protein also has a trans-activating activity, enhancing transcription on its own promoter and cellular promoters as well, to an extent that still merits investigation. Finally, a single pre-mRNA is processed to yield the complete set of mature mRNAs by a regulated combination of cleavage–polyadenylation and splicing events, depending both on the onset of replication of the viral genome and on cellular factors, not yet completely characterized although possibly, at least in part, specific to the erythroid lineage [35,36]. As a result, once macromolecular synthesis can start on an active dsDNA template, a biphasic pattern of viral expression is observed, an early phase antecedent to amplification of the genome, mainly leading to the production of the NS1 protein, and a later, leading to the production of structural VP proteins, and of the 11 kDa protein [37,38].

The effect of a productive replication in erythroid progenitor cells is cell death, which translates into a temporary and reversible block of erythropoiesis. The virus is able to modulate the cell cycle progression arresting cells in a late S/G2 phase, to its own advantage, mainly by the NS1 protein and its ability to interfere with the ATR-Chk1 pathway [39] and the deregulation of activity of E2F proteins [40]; the virus induces a DDR response, although dispensable for viral replication; the virus induces apoptosis [41], by its NS1 and 11 kDa proteins [42]. On the other hand, induction of inflammatory or necrotizing phenomena is not a common finding in infected bone marrow—with the exception of the rare condition of bone marrow necrosis observed in subjects predisposed to hematological disorders [43].

This scenario changes considerably when analyzing the interaction of B19V with cell types different from erythroid progenitors, which are normally non-permissive to viral replication, and where the outcome of infection is more variable and clinically heterogeneous [13]. A common theme is the persistence of the virus, or at least viral DNA, in disparate tissues, from skin to joints, heart, liver, spleen, and bone marrow, among others [44]. The frequency of detection usually is within 25–30% of positivity, not differing significantly between diseased or normal tissues, thus posing critical problems of interpretation, and assessment of any pathogenetic role and mechanisms of disease. Other than EPCs, experimental infection and evaluation of viral activity have been carried out for bone-marrow- and placental-derived mesenchymal stromal cells [45], endothelial cells of different anatomical districts [46,47], and normal or diseased fibroblasts [47,48]. All of these cellular elements constitute a diffuse reservoir for the virus, allowing for its persistence in the host, and posing a question on the actual possibility of B19V giving persistent infections and possibly reactivating from a quiescent state in a likely lifelong interaction. In addition, B lymphocytes from tonsillar tissues have been shown to harbor the virus and have also been tested in experimental infection [49]. Common to these systems are an internalization pathway not involving the VP1u binding to its cellular receptor, and the inability of the virus to complete a replicative cycle with the production of infectious progeny. An antibody-dependent enhancement (ADE) phenomenon has been described in endothelial cells, via the C1q receptor [50], B-cells, via the Fc receptor [49], and in the macrophage cell line U937 [51], but the increased internalization does not lead to a productive replicative cycle. Following internalization, the degree of expression of the viral genome in different cell types may vary. While in the more undifferentiated mesenchymal stromal cells (MSC) and in B-cells the genome appears silent, a basic transcriptional activity can be detected in endothelia and fibroblasts [47], with the opportunity for the virus to synthesize proteins such as the NS1. While insufficient for priming replication of the genome, NS1 protein may impact the cell phenotype, mostly inducing the expression of proinflammatory genes. In fact, the pathogenesis of B19V other than on erythropoiesis is linked to inflammatory and necrotizing phenomena, such as seen in arthritis, vasculitis, myocarditis, and other atypical manifestations, described at lesser frequencies in most body tissues. In the longer term, viral genomes will be silent, and be present in tissues at low copy numbers and absence of transcriptional activity, a phenomenon possibly subject to epigenetic regulation [52]. Thus, an abundance of viral genomes and the presence or absence of transcription may be assumed as a discriminating criterion for the involvement of the virus in pathogenesis, for example in the controversial role of B19V in dilated inflammatory cardiomyopathies [53].

The last contribution to the pathogenesis of B19V is determined by the extent and characteristics of the immune response, either for controlling the course of infection or as a contributor to immune-mediated pathogenetic mechanisms. The interaction of B19V with innate immunity sensors and effectors is not well characterized, though it constitutes a relevant research theme; the production of cytokines in the initial phase of infection is a known event, contributing to the first, prodromal phase of the clinical course of infection [54]. Adaptive immunity is better appreciated, less in the definition of cell-mediated immunity rather than the production of antibodies. While the former is at the basis of the sustained immune response against the virus, the latter is the correlate of protection that is normally assessed in clinical settings. Neutralizing epitopes have been mapped on the capsid shell, mostly on the common VP1/2 region and conformational [55,56], but a special emphasis has been granted to antibodies against the RBD in the VP1u region, recognizing linear epitopes [57]. Adaptive immunity is presumed to be lifelong and protective, although the efficacy of neutralization varies, and non-neutralizing immune responses have been observed, not only in immunodeficient subjects. On the whole, the adaptive immune response appears oligoclonal, and its characteristics determine whether the effective rapid or delayed clearance of the virus from the bloodstream will be obtained. In this context, autoimmune phenomena can occur. The production of autoreactive antibodies in the course of B19V infection is a well-described phenomenon [58,59], and although normally transitory, can critically contribute to systemic, rheumatic diseases.

## 4. B19V and Rheumatic Diseases

Joint involvement is a typical manifestation of B19V infection in adults and is very soon recognized [60,61]. It affects adults and women at a higher frequency, normally involves small joints in a symmetric fashion, can be very severe and debilitating, and has a chronic course. Synovia are infiltrated with inflammatory cells, and while synoviocytes do not support viral replication [62], the virus or its components can be detected in infiltrating monocytes or lymphocytes [63,64]. Symptomatology at the outset can be severe and a differential diagnosis with more severe pathologic conditions can be required; in the case of B19V-related arthritis, resolution of inflammation is the rule without permanent sequelae. The proposed pathogenetic mechanism includes activation of synoviocytes [65], possibly through the viral-associated Phospholipase A2 (PLA2) activity [66], as well as induction of an inflammatory local response possibly through an ADE-mediated mechanism and stimulation of monocytes and infiltrating lymphocytes.

A systemic inflammation, common in the initial prodromic phase of the infection, can rarely persist leading to a chronic syndrome with characteristics of chronic fatigue syndrome [67]. Persistent viremia and sustained production of proinflammatory cytokines in the context of a predisposed genetic background has been proposed as a pathogenetic mechanism related to B19V-induced chronic fatigue syndrome (CFS); treatment with immunoglobulin has been reported as effective [68], due to the immunomodulating properties of intravenous immunoglobulins (IVIG) other than their viral neutralization. However, a note of caution has been introduced after observation of a paradoxical, exacerbating effect, possibly due to the induction of an ADE phenomenon [69].

Several reports suggest that B19V could be involved in the pathogenesis of some autoimmune rheumatologic diseases such as rheumatoid arthritis (RA), juvenile idiopathic arthritis (JIA), systemic sclerosis (SSc), systemic lupus erythematosus (SLE) or vasculitis [70]. Among the most documented mechanisms involved in B19V-associated autoimmunity are molecular mimicry [59,71], B19V-induced apoptosis leading to T lymphocyte activation by self-antigens [72,73], and the phospholipase A2-like activity of B19V VP1u region [9,74], which may contribute to the inflammation and autoimmune diseases by the production of leukotrienes, prostaglandins, and cleavage products able to induce anti-phospholipid antibodies [70,75,76,77,78].

## 5. B19V and Rheumatoid Arthritis (RA)

Rheumatoid arthritis is a chronic systemic inflammatory autoimmune disease, characterized by symmetrical, destructive polyarthritis, systemic inflammation, and production of a variety of autoantibodies [79].

An association between B19V infection and RA was initially suggested since B19V arthritis often meets clinical diagnostic criteria for RA [60] and is sometimes followed by the appearance of rheumatoid factor (RF) [80]. Moreover, B19V DNA is frequently detected in the synovia of RA patients [81]. Currently, there are conflicting data regarding the etiologic association of B19V infection with RA. A number of studies did not find significant differences in the prevalence of B19V markers between RA patients and controls [82]. On the other hand, a recent study aimed to determine the prevalence of B19V infection in patients with RA compared to healthy controls and to assess its possible association with disease activity or severity, demonstrated that B19V DNA and an-ti-B19V IgG (but not IgM) was significantly higher in RA patients than healthy controls (*p* = 0.003 and *p* = 0.04, respectively) [83]. It was concluded that B19V infection might have a role in the etiopathogenesis of RA but not in disease activity or severity.

Takahashi and colleagues [84] found B19V DNA in synovium in 30 of 39 patients with RA, 4 of 26 patients with osteoarthritis, and 5 of 31 patients with trauma. Viral VP1 protein was detected in 27 patients with RA with active synovial lesions, but not in controls. Infectious virus was demonstrated in T and B lymphocytes, macrophages, and follicular dendritic cells. The presence of the virus was associated with increased IL-6 and TNF-α production [84].

It was demonstrated that the introduction of the non-structural protein 1 (NS1) gene of B19V could cause mice to be susceptible to polyarthritis through the upregulation of inflammatory cytokines, which are characteristic of RA lesions [85]. Moreover, immune responses to the B19V NS1 protein were associated with more severe courses of B19V infection [86] and with chronic B19V arthritis [87].

Exploratory factor analysis showed a correlation between the disease activity of patients with RA and the expression of B19V markers. The highest level of RA disease activity occurred in patients with active B19V infection [88].

Naciute and colleagues [89] studied the possible involvement of B19V infection in RA pathogenesis by investigating the presence of B19V infection markers (viral DNA and virus-specific antibodies) in association with the level of cytokines and RA clinical activity and aggressiveness. They found that B19V DNA prevalence was higher in patients with RA (25.4%) than in healthy controls (18.4%) and that B19V DNA in cell-free plasma (B19+p) was detected significantly often in RA patients in comparison with healthy controls (13.6% vs. 2%; *p* = 0.0002). 

A B19+p patient presented with higher disease activity and aggressiveness, decreased hemoglobin, and increased erythrocyte sedimentation rates. Moreover, IL-6 levels were significantly higher in RA patients than in healthy controls. Within the RA patients’ group, the IL-6 level was significantly increased in B19+p patients with disease activity scores of DAS28 > 5.2, high C-reactive protein, and low hemoglobin. Contrary to the healthy controls, the majority of RA B19+p patients lacked antibodies to VP-1S (VP1u) and VP-N, which correspond to the epitopes of neutralizing antibodies. Based on these results the authors concluded that B19V infection at least in some patients could be involved in RA pathogenesis.

## 6. B19V and Juvenile Idiopathic Arthritis (JIA)

Juvenile idiopathic arthritis includes a number of chronic rheumatic heterogeneous diseases, sharing chronic inflammatory arthritis of more than 6-week duration, of unknown origin. The last classification system proposed by the International League Against Rheumatism (ILAR) distinguishes Systemic JIA, RF-positive JIA, enthesitis/spondylitis-related JIA, early-onset ANA, positive JIA, other JIA, and unclassified JIA. A variety of autoantibodies including RF, anti-collagen II, and ACPA (anti-citrullinated protein antibodies) are produced by JIA patients [90]. 

The association between JIA and B19V infection was first suggested based on the similarities of clinical manifestations. Moreover, the onset of JIA was observed in patients with acute B19V infection [91].

The prevalence of B19V DNA in patients with JIA compared to controls was high (48% vs. 0%) [92]. In some cases, B19V infection has also been shown to precede the development of JIA and to induce antiphospholipid antibodies [93].

## 7. B19V and Systemic Sclerosis (SSc)

Systemic sclerosis is a chronic autoimmune rheumatic disease characterized by early endothelial damage and immunological abnormalities with progressive fibrosis in multiple organs, including skin, heart, vessels, and lungs. The endothelial dysfunction plays a primary role in the initiation and perpetuation of vasculopathy associated with SSc [94,95]. The subsequent fibroblast activation causes an excessive extracellular matrix (ECM) deposition and extended tissue fibrosis, the main process leading to organ failure [96]. The triggering factors of SSc remain unknown. In addition to the host’s genetic predisposition, several environmental agents have been considered, including toxic and infectious agents [96].

In 1999 Ferri and colleagues observed a higher prevalence of B19V viremia among patients affected by SSc, compared to healthy subjects [97]. This finding prompted further investigations on the possible role of this virus in the pathogenesis of scleroderma. Subsequent ex vivo studies demonstrated that persistent B19V infection was more frequent in scleroderma patients than in controls. In this case, viral DNA was detected in the bone marrow (which represents the main target of the virus) and/or in the skin (the typical target of the disease) of anti-B19 IgG-positive patients [98,99,100]. Moreover, in scleroderma patients, the prevalence of anti-B19V NS1 antibodies, previously suggested as a marker of persistent infection, was higher than in the control group [86,87]. Afterward, analysis of the skin biopsies from SSc patients revealed that B19V DNA and B19V mRNAs could be found in fibroblasts and in perivascular inflammatory cells. Moreover, in B19V-infected cells, tumor necrosis factor alpha (TNF-α) mRNAs were also detected. The degree of B19V mRNA expression correlated with endothelial cell degeneration and inflammation. These results suggested that B19V could be involved in the endothelial cell dysfunction [101]. It has also been demonstrated that B19V DNA can persist in SSc dermal fibroblasts propagated in vitro [48].

Further in vitro studies provided evidence that B19V can infect the target cells of SSc such as dermal fibroblasts [47,102], endothelial cells originating from various tissues [47,50], and different cell types belonging to the heterogeneous group of bone marrow-derived circulating angiogenic cells (CACs) [103], essential for vascular regeneration. None of these cell types resulted in permissive viral replication since no viral particles were produced. In the infected cells, only a partial expression of the viral genome was observed but not genome replication. 

Little is known about the consequences of abortive B19V infection in these cells. To demonstrate the possible role of B19V in scleroderma pathogenesis it is necessary to understand if the virus can induce functional alterations of infected cells and contribute to inflammation, fibroblast dysfunction, or defective endothelial cell homeostasis. Some recent in vitro investigations focalized on this aspect. For instance, Arvia et al. [102] showed that B19V infection can activate in vitro normal human dermal fibroblasts (NHDFs), increasing their ability of both migration and invasiveness and expression of mRNA of different profibrotic genes (alpha-smooth muscle actin (α-SMA), endothelin-1 (EDN-1), interleukin-6 (IL-6), transforming growth factor (TGF-β1 receptors 1 and 2), collagen chain 2 (Col1α2)), of some genes associated with inflammasome platform (absent in melanoma 2 (AIM2), interferon-inducible protein 16 (IFI16), interleukin 1 beta (IL-1β), caspase-1 (CASP-1)) and genes for some metalloproteases (MMP 2, 9 and 12). Since it has been demonstrated that MMP12 overexpressed by scleroderma fibroblasts can induce impaired human microvascular vein endothelial cells (HMVECs) proliferation, invasion, and capillary morphogenesis [104,105], B19V infection of normal human dermal fibroblasts (NHDF) could contribute to the defective angiogenic process, a pathological hallmark of SSc, responsible for the capillary loss and the subsequent ischemic organ injury [102].

In a subsequent study, we showed that in vitro infected cells develop typical senescence features like that observed in SSc dermal fibroblasts: enlarged and flat-shaped morphology, senescence-associated beta-galactosidase (SA-β-gal) activity and senescence-associated secretory (SASP)-like phenotype characterized by mRNA expression and release of some pro-inflammatory cytokines, along with activation of the transcription nuclear factor NFk_B_. The demonstration of the ability of B19V to activate dermal fibroblasts and to induce its senescence suggests that the virus may have a role in the pathogenesis of fibrotic lesions [106]. In fact, some previous studies suggested that cellular senescence has a role in the fibrotic process, such as that occurring in aging or SSc [107]. Indeed, SSc-associated fibrosis shares some pathological features with other age-related fibrotic diseases, such as idiopathic pulmonary fibrosis (epithelial and endothelial injury, immune dysregulation, and fibroblast activation with increased deposition of extracellular matrix) [108]. Furthermore, several studies have shown that advanced age is associated with a higher incidence and more severe disease and with increased mortality of SSc patients [109], suggesting the association of SSc with aging. Moreover, a higher SA-β-gal expression in SSc skin than in healthy skin was detected. Likewise, cultured fibroblasts from scleroderma patients showed higher SA-β-gal expression than control fibroblasts. Using a proteomic approach Dumit and collaborators [110] detected numerous age-dependent differences between normal and scleroderma fibroblasts, including the accumulation of SA-β-gal and evidence indicative of cellular senescence in the case of scleroderma fibroblasts. We also demonstrated by comet assay, that a subpopulation of fibroblasts from B19V-infected cultures showed a significantly higher level of DNA strand breaks and oxidative damage compared to mock-infected cells suggesting, that B19V can induce DNA damage in in vitro infected fibroblasts. An increased level and nuclear localization of ϒH2AX, a distinctive sign of DNA damage response, were also observed [106]. B19V-induced senescence and production of SASP-like factors in normal dermal fibroblasts could represent a new pathogenic mechanism of non-productive B19V infection, which may induce the fibrotic process.

In a recent study, we addressed the question of whether the fibroblast activation could be attributed to the activity of nonstructural (NS1) viral protein (unpublished data). The NS1 protein has a role in viral DNA replication, can modulate the expression of different cellular genes, and is known to induce alterations in the cellular environment linked to the pathogenesis of some B19V-associated diseases [3]. It has been demonstrated that NS1 protein trans-activates IL-6 promoter and induces increased expression of IL-6 in human hematopoietic cell lines K562, Raji, and THP-1 [111]. Upregulation of TNF-α in promonocytic cell line U937 has also been documented [51]. Furthermore, NS1 protein also induces expression of IL-1β and IL-18 through activation of caspase-1-associated NLR family pyrin domain containing 3 NLRP3 inflammasome in peripheral blood mononuclear cells (PBMCs) from Still’s disease patients [112]. To answer our question, primary cultures of NHDF were transfected with a B19V genomic insert excised from plasmid pCH10-pAs1, a minigenome maintaining replicative activity and expressing only the NS1 protein [113], and the expression of relevant selected cellular genes was analyzed by real-time RT-PCR as previously described [102]. In transfected fibroblasts, as compared to mock-transfected, we found significant increases in mRNA for IL-6, IL-1β, for some genes associated with inflammasome platforms such as Casp-1, IFI16, AIM2, and genes for MMP9 and MMP12. These results suggest that the upregulation of such genes in human fibroblast cultures could be stimulated by the NS1 protein. On the opposite, the levels of mRNA for α-SMA, END-1, TGFβ1, tumor growth factor TGFβ1 R1, TGFβ1 R2, NLRP3, connective tissue growth factor (CTGF), CoL1a2, MMP2 which resulted upregulated in B19V-infected fibroblasts [102], remained stable in transfected cultures (*p* ≥ 0,05). The expression of these genes could thus be stimulated by other viral components (unpublished data). Identification of the NS1 protein as a potential inducer of the expression of some profibrotic factors adds new light on the possible role of this protein in the skin fibrosis process.

The observation that B19V infection is associated with impaired endothelial regeneration through induction of apoptosis and dysregulated trafficking of infected circulating angiogenic cells (CACs) suggested that B19V infection can contribute to dysfunctional endogenous vascular repair [103,114,115]. The B19V-induced apoptosis of CACs, through some viral proteins (NS1, VP1u, and 11 kDa), was due to downregulation of BIRC3 (cellular inhibitor of apoptosis-2 (cIAP2), a potent suppressor of apoptotic cell death activation of caspases-8 and -10 [115] while the enhanced migration of CACs was associated with increased expression of surface CXCR4 [114].

It is known that abnormal innate immune response plays a role in the pathogenesis of systemic sclerosis [116]. It has been demonstrated that circulating mononuclear cells from patients with scleroderma, produce higher levels of cytokines and molecules involved in inflammation and fibrosis, compared to that from healthy subjects [117,118]. Moreover, different studies suggested the role of the inflammasome in the fibrotic process [119,120]. In fact, the upregulation of secretion of at least 40 genes involved in the inflammasome pathway and NLRP3-mediated secretion of IL-1β and IL-18 has been demonstrated in scleroderma fibroblasts [121]. There are many viruses able to activate inflammasomes [122,123,124], and it was hypothesized that persistent infection and chronic inflammasome activation are involved in the pathogenesis of SSc [121]. In vitro studies demonstrated that B19V abortive infection induces NLRP3-dependent caspase-1 activation and caspase-1 mediated IL-1β secretion in monocytic phorbol 12-myristate 13-acetate PMA-differentiated THP-1 cells. Moreover, cultured monocytes derived from scleroderma patients showed increased TNF-α production, NLRP3 expression, and caspase-1 activation following B19V infection [125].

Based on these data, it can be hypothesized that the abortive B19V infection and fibroblast alteration observed in vitro may also occur in vivo, either in the case of primary B19V infection or in the case of expression of the persistent virus due to changes in the cellular environment or to co-infection with other viruses [126,127].

## 8. B19V and Systemic Lupus Erythematosus (SLE)

Systemic lupus erythematosus (SLE), an autoimmune and multi-systemic disease characterized by inflammation and abnormal production of autoantibodies, can affect various organs, including skin, joints, lungs, kidneys, and the nervous system, with a chronic relapsing/remitting course. The mechanism that triggers the aberrant innate and adaptive immune response is still not well known [128]. Many viruses such as Epstein–Barr virus (EBV), parvovirus B19 (B19V), human endogenous retroviruses (HERVs), and cytomegalovirus (CMV) have been linked to the pathogenesis of SLE in genetically predisposed patients [129].

The association between B19V infection and SLE is based on some clinical, laboratory, and epidemiological data. For instance, there are some indications, that SLE can develop following B19V infection [130] and that B19V infection coincides with an aggravation of SLE [131,132]. Moreover, the clinical presentation and laboratory findings identical to those in lupus can appear in patients with acute B19V infection.

It was also demonstrated that B19V-infected patients produce different autoantibodies including antinuclear antigen antibodies (ANA), anti-double stranded DNA (dsDNA), rheumatoid factor (RF), and anti-phospholipid (APL) antibodies [133,134,135], commonly found also in patients with SLE. In fact, it is known that some anti-B19V antibodies to the capsid proteins can interact with several host antigens including keratin, collagen 2, cardiolipin, and ssDNA [59]. In addition, B19V induces strong apoptosis of bone marrow erythroid precursors, leading to the release of numerous altered self-antigens [136,137] and subsequent production of autoantibodies.

There is accumulating evidence that B19V has a role in the pathogenesis of antiphospholipid syndrome (APS) characterized by the production of abnormal antibodies called antiphospholipid (aPL) antibodies and associated with a higher risk of thrombosis and/or pregnancy morbidity. It can be either primary or developed within the setting of SLE (secondary APS) [138]. In fact, a higher prevalence and higher titer of anti-B19V IgM and IgG were found in a subset of SLE patients with APS compared to those without APS, suggesting a link between infection and APS [139]. The role of VP1u protein as an antigen-triggering APS-like syndrome has been established [140].

Some studies also indicate that B19V is associated with dilated cardiomyopathy (DCM), a specific SLE complication. In fact, the prevalence of anti-B19-NS1 and anti-B19-VP1u IgG was significantly higher in patients with DCM than in the general SLE population [141].

## 9. B19V and Vasculitis

Vasculitic diseases are characterized by inflammation and destruction of blood vessels and the appearance of a variety of autoantibodies, including anti-glomerular basement membrane (anti-GBM) antibodies, antineutrophil cytoplasmic antibodies (ANCAs) directed at proteinase-3 (PR3) and myeloperoxidase (MPO), and anti-endothelial cell antibodies [142].

In general, an association between B19V infection and different vasculitic diseases has been proposed, mainly based on small case–control series or case reports. However, contrasting reports, the lack of a larger study series, and the demonstration of plausible pathogenetic mechanisms presently prevent a firm assessment of the role of B19V in this group of diseases.

An association between B19V infection and Kawasaki disease was first proposed, based on a higher incidence of infection in a small group of pediatric patients [143]; however, this finding was not immediately confirmed [144]. An active B19V infection can be diagnosed in patients whose clinical presentation is compatible with Kawasaki disease, or concomitant to underlying Kawasaki disease, but an etiological link is presently unsupported by experimental evidence [145,146].

A similar uncertainty is pertinent to a causal association between Henoch Schonlein purpura and B19V infection, where opposite to single case reports demonstrating active infection and presence of a viral component in endothelial cells, such as in [147], the only available study based on a case–control series fails in establishing a higher viral incidence [148] as a basis for an etiological role of B19V. Further, case reports assessing a role for B19V, and contrasting studies denying it, are present in the literature for other vasculitides such as polyarteritis nodosa [149,150]; Behcet’s disease [151,152]; and Wegener’s granulomatosis [153,154].

In summary, the literature on the involvement of B19V in vasculitic diseases is limited and not conclusive. While case–control prevalence studies usually indicate a lack of association, an etiological role in single case reports is mostly supported by the direct detection of the viral genome or viral capsid proteins in the diseased tissues. In these cases, a plausible mechanism of disease involves infection of endothelial cells virus-induced inflammation, and development of an immune system response with production of autoantibodies and activation of cell-mediated immunity. Given the rarity of these clinical presentations, the host genetic and immunologic background of the host likely plays a major role, but any hypothesis is presently lacking experimental support. Further research is required to investigate the role, if any, of B19V in this group of diseases.

## 10. Conclusions

On clinical-translational grounds, rheumatological diseases pose a challenge for diagnosis, etiological clues, and treatment options. Concerning B19V, the commonly available diagnostic tools consent discrimination of recent or active infection mainly based on the detection of specific IgM antibodies and/or the presence of viral DNA in peripheral blood, by qPCR assays [155]. However, to associate B19V to rheumatic diseases as discussed in this review, these diagnostic criteria may not directly lead to a conclusive etiological diagnosis. In fact, both a superposition of a B19V infection to an unrelated, underlying disease, or on the contrary lack of inclusion of B19V in the diagnostic workup can lead to diagnostic pitfalls. Increased awareness of the role of B19V in rheumatic diseases and appropriate use of diagnostic tools is thus justified. Notably, assessment of a role of B19V in the pathogenesis of rheumatic diseases will necessarily require investigation of biopsies for the detection of viral markers in the context of pathological tissues, as a basis for a causal link of virus and disease. In situ detection of viral DNA, viral mRNAs and viral proteins can provide justification for an etiological link to observed pathological processes [13]. Treatment options to B19V are limited, although research on antiviral strategies is now starting to provide some results [156,157]. In case of rheumatologic diseases, the choice of treatment is guided by the disease and not by any possible role of the virus. Thus, unraveling the pathogenetic mechanisms of B19V in rheumatologic diseases may provide identification of targets suitable for the development of specific novel antiviral drugs or therapeutic choices.

## Figures and Tables

**Table 1:** Clinical manifestations of parvovirus B19 infection.

Category	Frequent	Sporadic
Hematological	Transient anemia Aplastic crisis Chronic anemia Chronic Pure Red Cell Aplasia	Bone marrow necrosis and fat embolism Myelodysplastic syndrome Thrombocytopenia/Granulocytopenia Pancytopenia Idiopathic thrombocytopenic purpura Hemophagocytic lymphohistiocytosis
Systemic/Rheumatological disease	Aspecific febrile illness Mono or polyarthritis Chronic arthralgias	Chronic fatigue syndrome Vasculitis Scleroderma Systemic lupus erythematosus Rheumatoid arthritis Juvenile idiopathic arthritis
Specific tissue/organ disease	Erythema infectiosum	Petechial purpura Papular purpuric gloves and socks syndrome Acute myocarditis/pericarditis Chronic inflammatory myocarditis Myositis Hepatitis Glomerulonephritis Meningitis/Encephalitis Peripheral neuropathy
Infection in pregnancy	Intrauterine infection Fetal anemia Fetal hydrops Fetal death	Mirror syndrome Meconium peritonitis Fetal malformations Congenital anemia

## Data Availability

Not applicable.
